# The Story of the Insane from Year to Year

**Published:** 1899-08-19

**Authors:** 


### THE STORY OF THE INSANE FROM YEAR TO YEAR.

(Eleventh Series.)

### LANCASHIRE COUNTY ASYLUM AT LANCASTER MOOR.

On the first day of 1897 this asylum contained close on 2,000 patients, and this number was exceeded by 68 on the last day of December. There were 433 first admissions, 42 readmissions, 144 discharged recovered, 67 discharged relieved, and 145 deaths. These numbers show that the recoveries were 30·32 per cent. of the admissions, and the deaths 7·11 per cent. of the average number resident. Of these deaths 36 were caused by cerebral and spinal diseases, 16 by pneumonia, 32 by consumption, 8 by enteritis, and 5 by enteric fever. Of the year's admissions 42 were driven insane by intemperance in drink, 55 by hereditary tendency, and 73 by previous attacks. There is always something in Dr. Cassidy's reports which affords food for thought, and it is rather alarming to find that in the year 1887 the ratio of the registered insane was 1 in 344 of the population, and that now it is 1 in 308; and Dr. Cassidy truly says that it is not comforting to be told that these figures merely show an accumulation of the insane and not an increase, because it is this very accumulation which constitutes the difficulty—a difficulty which he says calls for a drastic remedy. Very good; but what is this remedy? We do not suppose that Dr. Cassidy has any more hope than have we that the course of insanity will be stopped by costly pathological investigations, but this very report shows that 97 of the year's admissions were due to drink and heredity. Here, then, are the enemies to attack. We regret that we have not space to quote Dr. Cassidy's remarks on the absurd reports which the Lunacy Commissioners now require after the escape of a patient; and while sympathising heartily with the "over-burdened medical superintendent," we fondly hope that the liberty of the patients will not be curtailed owing to these and many other useless documents. Dr. Harbinson, the senior medical assistant, retired owing to ill-health, and received a superannuation allowance; and Dr. Cassidy pays a well-merited tribute to his former colleague, who was veritably a "kind-hearted, conscientious, and devoted assistant." The weekly maintenance rate was 8s. 2⅝d. per head.

### SURREY COUNTY ASYLUM AT BROOKWOOD.

There were 1,059 patients in this asylum on the first day of January, 1897, and on the last day of the year the number was exactly the same. The first admissions during the year were 252, the readmissions 44, the recoveries 82, and the deaths 90. The recoveries stand at 28·47 per cent. of the admissions, and the deaths at 8·41 on the average number resident. Of these deaths 42 were caused by cerebral and spinal diseases, 8 by pneumonia, 13 by consumption, and 2 by enteric fever. Eighty of the admissions owed their attacks of insanity to such causes as domestic troubles, mental anxiety, religion, &c.; 41 to intemperance in drink, 58 to hereditary influence, 7 to epilepsy, and 5 to influenza. The asylum was overcrowded during the year, and a new asylum for the county is spoken of. As already stated, there were two deaths from enteric fever, and there were two other cases which ended favourably, one of them being the temporary assistant medical officer. The origin of the diseases could not be traced, and it is noteworthy that sporadic cases of this disease often make their appearance in asylums and baffle the most careful investigation of their origin. Dr. Barton states that neither restraint nor seclusion was found necessary during the year; but the question of restraint or non-restraint, of seclusion or non-seclusion, is by no means always one of necessity; but it is one of desirableness. Is it better for the patient himself and for the asylum generally that he should be restrained or secluded? We believe that the tendency in the future will be towards giving enormously increased freedom to the majority of patients in our county asylums, coupled with a diminished amount of freedom to the very few. In this report we read that a homicidal assault was committed by an ex-criminal patient on an attendant, and that the latter was unfit for duty for some days. There are two assistant medical officers, and the commissioners recommend the appointment of a third one. The weekly charge for maintenance for in-county patients is 10s., and for out-county patients 14s. and 15s.

### THE MAPPERLY HILL ASYLUM, NOTTINGHAM.

In this asylum the number of patients increased by 13 during the year 1897. There were 134 first admissions, 26 readmissions, 64 recoveries, 8 discharged relieved, and 52 deaths. The recoveries reached 42·1 per cent. on the admissions, which is rather higher than the county asylum average, and also a little above their own average. The deaths stand at 8·6 per cent. calculated on the average number resident, and this is below the average. On Table X. we find that the most potent moral cause of insanity among the year's admissions was mental anxiety, which accounted for 20; and among the physical causes intemperance and heredity have, as usual, an unenviable precedence, the former for 34 and the latter for 12. "Previous attacks" account for 31, and no doubt many of these should be classed in one or other of the two first-named. The asylum is again being added to, and as it already contains 614 patients it must be rapidly approaching the limits beyond which it is not desirable to go. Mr. Powell says that his nurses and attendants have discharged their duties very satisfactorily, but he regrets the frequent changes among the nursing staff. He is not the only superintendent filled with similar regrets, and we feel sure that these changes are practically inseparable from asylum service, and deplorable as are these frequent changes they are preferable to much prolonged service. At present asylum affairs are in an unsettled state, and will be until Parliament settles the pensions question in a liberal spirit. If this were done, and if the various committees slightly, even, raised the wages and somewhat improved the nurses' surroundings, there is almost a certainty that suitable people would come forward. Mr. Powell, assisted by Dr Vincent and Dr. Montgomery, continue to instruct the staff in matters connected with nursing, and two of the attendants passed the Medico-Psychological Examination, and obtained their certificates. The weekly charge to the union is 10s. per head.

